# HIV-related public stigma in the era of “Undetectable = Untransmittable”: a population-based study in Hong Kong

**DOI:** 10.1186/s12889-024-18974-0

**Published:** 2024-06-06

**Authors:** Leonia Hiu-Wan Lau, Man-Po Lee, Bonnie Chun-Kwan Wong, Tsz-Shan Kwong, Wai-Man Hui, Jacky Man-Chun Chan, Shui-Shan Lee

**Affiliations:** 1 School of Nursing, Tung Wah College, Hong Kong, China; 2 Department of Medicine, Queen Elizabeth Hospital, Hong Kong, China; 3 Department of Health, Centre for Health Protection, Hong Kong Special Administrative Region Government, Hong Kong, China; 4 Department of Medicine, Alice Ho Miu Ling Nethersole Hospital, Hong Kong, China; 5 Department of Medicine and Geriatrics, Princess Margaret Hospital, Hong Kong, China; 6 Stanley Ho Centre for Emerging Infectious Diseases, The Chinese University of Hong Kong, Hong Kong, China; 7 S.H. Ho Research Centre for Infectious Diseases, The Chinese University of Hong Kong, Hong Kong, China; 8 Postgraduate Education Centre, Stanley Ho Centre for Emerging Infectious Diseases, Prince of Wales Hospital, Hong Kong, China

**Keywords:** HIV, People living with HIV, Stigma, Public stigma, Latent class analysis

## Abstract

**Background:**

While global efforts are increasingly relying upon biomedical advancements such as antiretroviral therapy and pre-exposure prophylaxis (PrEP) to end the HIV epidemic, HIV-related stigma remains a concern. This study aimed to assess the general public’s awareness and perception of “Undetectable = Untransmittable” (U = U) and PrEP, and the patterns of public stigma towards people living with HIV (PLWH) and their determinants in an Asian Pacific city.

**Methods:**

A population-based, self-administrated online survey was conducted between 10–20 March 2023. All adults aged ≥ 18 years and currently living in Hong Kong were eligible. Participants’ socio-demographic characteristics, awareness and perception of U = U and PrEP, as well as HIV-related stigma drivers, experience and practices were collected. Latent class analysis was used to delineate population subgroups based on their stigma profiles as reflected by 1.) fear of infection, 2.) concern about socioeconomic ramification of the disease, 3.) social norm enforcement, 4.) perceived stigma in the community, and 5.) stigmatising behaviours and discriminatory attitudes. Memberships of identified subgroups were then correlated with sociodemographic factors, awareness and perception of U = U and PrEP, using multinominal logistic regression.

**Results:**

Responses from a total of 3070 participants (55% male; 79% aged 18–54) were analysed. A majority, 69% and 81%, indicated that they had never heard of U = U and PrEP respectively, and only 39–40% of participants perceived these to be effective in protection from HIV. Four distinct subgroups were identified, namely *“Low stigma”* (37%), *“Modest stigma”* (24%), *“Moderate stigma”* (24%), and *“High stigma”* (15%). Compared with *“Low stigma”*, lack of awareness of and/or negative perceptions towards U = U and/or PrEP, not knowing any PLWH were associated with increased odds of higher stigma group membership. Lower educational level and not in employment were associated with increased odds of membership in *“Moderate stigma”* and *“High stigma”.* While older people were more likely to belong to *“High stigma”,* female were more likely to belong to *“Moderate stigma”*. *“Modest stigma”* included more younger people who were economically active.

**Conclusion:**

Two-thirds of participants endorsed modest-to-high HIV-related stigma, suggesting the prevalence of HIV-related stigma was high among the general population in Hong Kong. Tailored interventions targeting specific stigma drivers and manifestations of individuals as reflected from the stigma profiles of distinct subgroups could form an important strategy for stigma reduction.

## Introduction

Recent advances in antiretroviral therapy (ART) have transformed human immunodeficiency virus (HIV) infection from a fatal disease to a chronically manageable condition. The introduction of ART-based treatment as prevention strategy (TasP) and pre-exposure prophylaxis (PrEP) have further advanced HIV prevention. TasP refers to the use of ART to reduce level of infectiousness in people living with HIV (PLWH). The explainer “Undetectable = Untransmittable” (U = U), has been put forward which, by translating the scientific evidence from TasP, serves to destigmatise HIV in the general population. It asserts that PLWH who achieve and maintain an undetectable viral load level (i.e., the viral load level is so low that it could not be detected by standard test) by consistent use of ART could not sexually transmit HIV to others. On the other hand, PrEP involves the use of antiretrovirals among HIV uninfected individuals in anticipation of potential HIV exposure to reduce the risk of infection. Accumulating evidence supports the key role of U = U and PrEP in mitigating HIV transmission and reducing HIV incidence [1–4]. While global effort has been increasingly relying upon U = U and PrEP to end the HIV epidemic, its success at population level could be hindered by HIV-related stigma as a major barrier to access to HIV testing, linkage to and retention in care, uptake and adherence to the treatment.

Stigma can be conceptualised as shaming, disgracing, and social discrediting of individuals with particular attributes, which involves a sociocultural process comprising of difference labelling, cognitive separation, stereotyping, negative emotion, status loss and social rejection [5, 6]. Within the context of HIV, stigma is often grounded on high level of self‐responsibility attached to HIV [7, 8]. PLWH are often viewed as deserving of HIV because of their “bad” or “risky” behaviours such as unsafe sex and intravenous drug use. HIV-related stigma is also related to misconceptions about HIV transmission and fear of infection through casual contact [7, 8]. HIV-related stigma could present itself in two forms, namely public stigma and self-stigma. Public stigma refers to the negative reactions held by members of the general public (i.e., those without HIV infection in the community) towards PLWH. Self-stigma is the product of public stigma, which entails awareness, internalisation, acceptance and application among PLWH of the negative beliefs and feelings associated with HIV, together with experience of the negative consequences (e.g., unfair treatment). Despite tremendous advancements and notable progress in HIV prevention in the era of U = U and PrEP, HIV-related stigma remains ubiquitous and pervasive across the world.

In Hong Kong, the ART coverage is high among PLWH while PrEP is generally not available. The HIV prevalence in Hong Kong is relatively low [9], yet public stigma toward PLWH remains prevalent [10, 11]. The blend of Chinese and Western cultures has further complicated the manifestation of public stigma. The “face” concern, culture expectation in continuation of family lineage, and moral standing embodied in Chinese culture could exacerbate the stigma towards PLWH [12–14]. To date, only limited studies have examined the public stigma among the general population in the Chinese contexts. Furthermore, majority of the research on HIV-related stigma have employed variable-centred approach, which measured and characterised the stigma by considering there was population homogeneity in regard to the related indicators. Such approach might not be sufficient to characterise the full picture of stigma which is complex and multidimensional in nature. Targeting the general population in Hong Kong, we set out to assess the awareness and perception of U = U and PrEP, to characterise the community patterns of HIV-related public stigma and to explore its correlations with sociodemographic factors, awareness and perception of U = U and PrEP.

## Methods

### Participants and procedures

A cross-sectional population-based survey was conducted in Hong Kong. The target population was citizens (i) currently living in Hong Kong, (ii) aged 18 years or above, and (iii) who were able to respond to an online questionnaire in Chinese or English. Participants were recruited through the Hong Kong Public Opinion Research Institute (HKPORI) to complete an online anonymous self-administered survey from March 10–20, 2023. Invitation emails with the survey link attached were sent to all current members of the PopPanel held by HKPORI. The PopPanel, a panel of the general public, included both probability based members who were randomly recruited through previous telephone surveys as representative of Hong Kong population, and non-probability based members who voluntarily joined through online registration. Given around 100,000 members under this panel, this panel provided a framework for sampling and conducting survey. With the assumption of a 3% margin of error, 95% level of significance, 50% of the population having certain knowledge or attitude, and 6,410,000 persons aged 18 years or above in mid-2022 in Hong Kong, we targeted to recruit 1067 participants. An incentive in the form of an HKD$25 voucher was given to first 500 participants upon completion of the survey for compensation of their time. Ethical approval was granted by the Survey and Behavioural Ethics Committee of the Chinese University of Hong Kong. Informed consents were obtained from all participants included in the study.

### Measurements

The questionnaire in the survey was formulated by the research team based on literature review. The questionnaire items were evaluated by a team of experts including experienced public health professionals and HIV specialists, and further piloted tested to ensure high face validity and content validity. The questionnaire comprised three sections including socio-demographic, HIV-related stigma and awareness and perception of U = U and PrEP.

#### Socio-demographics

Socio-demographic characteristics were captured with the following variables: age, gender, education attainment, employment status, personal monthly income, ethnicity and residency status, marital status, and is one knows someone with HIV.

#### HIV-related stigma

Measurement of HIV-related stigma was guided by the Health Stigma and Discrimination (HSD) Framework which delineates the stigmatisation process in the context of health [15, 16]. Based on the HSD framework, together with literature review and expert consultation, a total of 26 items were developed to capture HIV-related stigma of the general population in five domains, including (1) *stigma drivers—fear of infection* (i.e., PLWH is perceived as threatening due to the infectious nature of HIV and misinformation about HIV transmission); (2) *stigma drivers—concern about socioeconomic ramification* (i.e., PLHV is perceived to have poor prospects in employment, friendship, romantic relationship and family life); (3) *stigma drivers—social norm reinforcement* (i.e., PLHV is perceived to be associated with socially and/or morally disapproved behaviours (e.g., unsafe sex, intravenous drug use)); (4) *stigma experiences* – *perceived stigma* (i.e., perception of stigma towards PLWH that exist in the community); and (5) *stigma practices—stigmatising behaviours and discriminatory attitudes*. Items were generally rated on a 6-point scale, while those assessing domain 5 were rated on a 5-point scale (Supplementary Table 1).

#### Awareness and perception of U = U and PrEP

Awareness of U = U and PrEP was assessed from responses to dichotomised questions “Have you ever heard about “U = U” / “PrEP” before?”. Following a brief description of U = U and PrEP, perceptions of U = U and PrEP were assessed by respondents’ level of agreement (on a six point scale, from strongly disagree to strongly agree) with statements reflecting scepticism towards U = U (e.g., “There is still risk of transmission even if the viral load of a person living with HIV is undetectable.”) and concerns about anticipated risk compensation associated with U = U (e.g., “U = U concept could lead to increased condomless sex.”). Respondents were also asked to rate their perceived impact of U = U in improving the comfort level with PLWH (no difference, more at ease, less at ease) as well as their perceived degree of protection (0–100%) offered by U = U, PrEP and other methods such as using condom (Supplementary Table 2).

### Statistical analysis

Descriptive analysis was performed to summarise respondents’ socio-demographic characteristics, awareness and perception of U = U and PrEP. Socio-demographics of respondents were compared to the 2021 population census in Hong Kong with Cohen’s w effect size (small: 0.1; medium: 0.3; large: > 0.5) to evaluate the representativeness of the study sample. Latent class analysis (LCA) was performed using the poLCA package in R software version 4.0.0 to delineate population subgroups based on their endorsement of HIV-related stigma drivers and manifestations [17]. LCA is a person-centred approach which identifies probabilistic homogenous subgroups within a heterogenous population based on their responses to a set of observed indicators [18], and in this case, the response to the 26 items measuring HIV-related stigma.

We started with two-class modelling with successive models fitted with increasing number of classes (up to five). The final model selection was based on a balance of 1.) lower Bayesian Information Criterion; 2.) entropy > 0.7 (indicating the ability of the models to accurately identify distinct latent classes); and 3.) clinical interpretability (whether each class could be given a meaningful label) [18]. Each respondent was then assigned to the latent class for which his/her membership probability was the highest. Multinomial logistic regression was performed to examine the relationship between socio-demographics, awareness and perception of U = U and PrEP and latent class memberships. In the data analysis, response categories for items measuring HIV-related stigma, awareness and perception of U = U and PrEP were collapsed and recoded into 2-level or 3-level variables for the purpose of easy interpretation (Supplementary Table 1 & 2). Statistical analyses other than LCA were performed using SPSS version 25 (SPSS Inc., Chicago, IL) with statistical significance defined by two-sided p values of less than 0.05. Complete case analyses were performed in addressing the missing data.

## Results

### Characteristics of study population

Email invitations were sent to 81,387 potential participants in the pool of HKPOP panel members, among which 3,181 who fitted the inclusion criteria had successfully completed the survey. After excluding 27 claiming that they might have already been HIV infected and 84 with incomplete data on latent class indicators (i.e., variables to be included in LCA), totally 3,070 respondents constituted the final sample for current analysis. Around half (55%) were male and three quarters (79%) aged 18–54, and only 6% reported to know someone with HIV. Overall, our study sample was reasonably representative of the Hong Kong general population, except that our sample tended to be younger and better educated **(**Table 1**).**

**Table 1:** Background characteristics of study sample and comparison against Hong Kong general population (*N* = 3070)

Socioeconomic characteristics	Study sample n (%)	HK Population ^a^ (%)	Effect size ^b^
**Gender**(missing = 29)			
Male	1680 (55.2)	45.6	0.19 ^c^
Female	1354 (44.5)	54.4	
Transgender	7 (0.2)	/	
**Age**(missing = 14)			
18–34	1060 (34.7)	32.2	0.34
35–54	1351 (44.2)	31.5	
55 or above	645 (21.1)	36.4	
**Education**(missing = 21)			
Secondary or below	415 (13.6)	68.8	1.19
Post-secondary	2634 (86.4)	31.2	
**Employment status**(missing = 32)			
Employed	2320 (76.4)	49.7	0.53
Economic inactive (e.g., employed, student, homemaker, retired)	718 (23.6)	50.3	
**Personal monthly income**(missing = 51)			
Lower (≤ median personal monthly income)	1010 (33.5)	/	Nil ^d^
Higher	2009 (66.5)	/	
**Ethnicity and residency status**(missing = 18)			
Chinese Hong Kong permanent resident	3025 (99.1)	90.9	0.12
Others (e.g., Chinese Hong Kong non-permanent resident / non-Chinese resident in Hong Kong)	27 (0.9)	9.1	
**Marital status**(missing = 17)			
Married or cohabiting	1432 (46.9)	52.2	0.11
Single/ divorced/ separated/ widowed	1621 (53.1)	47.8	
**Knows someone with HIV**(missing = 75)			
Yes	190 (6.3)	/	Nil ^d^
No	2805 (93.7)	/	

^a^Based on 2021 Hong Kong Population Census (Census & Statistics Department HKSAR)

^b^Cohen’s effect sizes *w* were calculated via the formula *w* = $\sqrt{\sum_{i=1}^{m}\frac{{((po\left(i\right)-p1\left(i\right))}^{2}}{po(i)}}$, where$po\left(i\right)$and$p1\left(i\right)$are the observed proportions in the i’^th^ category from the census data and survey data respectively

^c^The “Transgender” category of the variable “Gender” was excluded from compassion as it was unavailable from 2021 census

^d^The variable “Personal monthly income” and “Knows someone with HIV” was excluded from comparison. as it was unavailable form 2021 census

### Awareness and perception of U = U and PrEP

Most respondents indicated that they had never heard of U = U (69%) or PrEP (81%) **(**Table 2**)**. They generally expressed scepticism towards U = U, of which 78% believed that there remained a transmission risk even when the undetectable viral load had been achieved; 86% agreed that even if the viral load of PLWH had reached a very low level, there was no guarantee of future fluctuations that would affect the risk of transmission; and 92% believed that the U = U status was largely dependent on the personal responsibility of PLWH in drug adherence. More than half of the respondents were concerned about the anticipated risk compensation associated with U = U, which could lead to increase in condomless sex (72%) or number of sex partners (52%). While more than three quarters (83%) believed that conventional prevention strategies such as condom use offered a lot to complete protection (i.e., 60% -100% protection), less than half believed that the same level of protection could be provided by achieving U = U with ART (39%) and using PrEP (40%). Regarding the perceived impact of U = U on acquaintances with PLWH, only about one quarter of the respondents indicated that this could make them more at ease working or attending class (28%) together; being friend and having social interaction (30%); having causal physical contact (31%) and developing intimate relationship (28%) with PLWH who is on ART. A small proportion believed that the U = U knowledge made themselves less at ease in getting along with PLWH, especially in developing intimate relationship (16%).

**Table 2:** Awareness and perception towards U = U and PrEP (*N* = 3070)

Variable	No. respondents	%
**Awareness of U = U**(missing = 26)		
Yes	933	30.7
No	2111	69.3
**Awareness of PrEP**(missing = 18)		
Yes	557	18.3
No	2495	81.3
**Scepticism towards U = U and anticipated risk compensation related to U = U**		
There is still risk of transmission even if the viral load of a person living with HIV is undetectable. (agree) (missing = 17)	2381	78.0
Even if the viral load of a person living with HIV has reached a very low level, there is no guarantee of future fluctuations that would affect the risk of transmission. (agree) (missing = 17)	2631	86.2
Whether people living with HIV can maintain U = U status depends largely on medication adherence. (agree) (missing = 14)	2802	91.7
U = U concept could lead to increased condomless sex. (agree) (missing = 20)	2185	71.6
U = U concept could lead to increased number of sex partners. (agree) (missing = 20)	1570	51.5
**Perceived effectiveness of different strategies**		
Using condom for sex (missing = 17)		
0%-60% (no protection at all to some protection)	532	17.4
61%—100% (a lot of to complete protection)	2521	82.6
Taking pre-exposure prophylaxis (missing = 29)		
0%-60%	1816	59.7
61%—100%	1225	40.3
HIV-positive partner taking drugs to maintain undetectable viral load (missing = 19)		
0%-60%	1871	61.3
61%—100%	1180	38.7
Reduction in number of sexual partners (missing = 19)		
0%-60%	1081	35.4
61%—100%	1970	64.6
**Impact of U = U in improving comfort level with PLWH**		
Work or attend class with him/her (missing = 7)		
No difference	2112	69.0
More at ease	870	28.4
Less at ease	81	2.6
Make friend with him/her, and have social interactions without physical contact (missing = 8)		
No difference	2056	67.1
More at ease	905	29.6
Less at ease	101	3.3
Have casual physical contact with him/her (missing = 6)		
No difference	1974	64.4
More at ease	945	30.8
Less at ease	145	4.7
Develop intimate relationship with him/her (missing = 6)		
No difference	1729	56.5
More at ease	859	28.0
Less at ease	476	15.5

## Delineation of community patterns of HIV-related stigma

A four-class model was determined to be the most parsimonious and provides informative explanation of the data **(**Table 3**)**. Class proportion and class-specific item-response probabilities of the latent class identified are summarised in Table 4.

**Table 3:** Fit statistics for latent class models with varying numbers of latent classes

Number of latent classes	AIC^a^	BIC^b^	Entropy^c^
2 classes	99653.38	100069.4	0.8137837
3 classes	97723.17	98350.23	0.8303097
**4 classes**	**96139.62**	**96977.71**	**0.8446884**
5 classes	95520.32	96569.45	0.8328952

^a^*AIC* Akaike Information Criterion, lower value of AIC indicates a better-fitting model

^b^*BIC* Bayesian Information Criterion; lower value of BIC indicates a better-fitting model

^c^Entropy > 0.7 indicating a good separation of classes

**Table 4:** Result of LCA – class proportion and class-specific probabilities

Latent class	1	2	3	4	Chi-square (*p*-value)
**Assigned label**	*Low stigma*	*Modest stigma*	*Moderate stigma*	*High stigma*	
**Class proportion**	0.37 (*n* = 1145)	0.24 (*n* = 736)	0.24 (*n* = 741)	0.15 (*n* = 448)	
**Item-response probabilities**					
**Drivers of stigma**					
**Fear of infection**					
**Endorsement of misconception about HIV transmission**					
Touching a person living with HIV	0.01	0.00	0.45	0.40	< 0.001^*^
Mouth to mouth kissing with a person living with HIV	0.44	0.46	0.98	0.82	< 0.001^*^
Sharing meals / eating utensils with a person living with HIV	0.09	0.09	0.81	0.57	< 0.001^*^
Sharing toilet or swimming pools with people living with HIV	0.15	0.15	0.89	0.63	< 0.001^*^
Mosquito bites	0.22	0.23	0.75	0.58	< 0.001^*^
**Expression of fear**					
HIV infection is a horrible illness	0.60	0.81	0.84	0.95	< 0.001^*^
**Comfort level (uncomfortable/ uneasy) with PLWH**					
Work or attend class with him/her	0.01	0.02	0.03	0.74	< 0.001^*^
Make friend with him/her, and have social interactions without physical contact	0.00	0.02	0.04	0.85	< 0.001^*^
Have casual physical contact with him/her	0.03	0.09	0.20	0.97	< 0.001^*^
Develop intimate relationship with him/her	0.65	0.93	0.90	0.99	< 0.001^*^
**Social norm enforcement**					
**Presence of stereotypes**					
Majority of people living with HIV in Hong Kong are men who have sex with men, illicit drug users, commercial sex workers or their patrons	0.38	0.52	0.58	0.77	< 0.001^*^
**Concern about socioeconomic ramification of the disease**					
**Perceived PLWH could not live their lives as other people**					
Working	0.00	0.09	0.05	0.15	< 0.001^*^
Social life	0.00	0.16	0.07	0.23	< 0.001^*^
Marriage	0.00	0.59	0.27	0.59	< 0.001^*^
Sexual life	0.08	0.96	0.53	0.72	< 0.001^*^
Having children	0.26	0.95	0.64	0.80	< 0.001^*^
**Manifestations of stigma**					
**Stigma experience—Perceived stigma in the community**					
A person living with HIV loses respect in the community	0.79	0.81	0.81	0.88	< 0.001^*^
People in the community often talk badly about people living with HIV	0.58	0.61	0.58	0.67	0.011^*^
**Stigma practices- Discrimination**					
PLWH deserve same level social support as those with other chronic illness					
Agree	0.79	0.61	0.63	0.54	< 0.001^*^
Not sure (slightly agree/ slightly disagree)	0.17	0.33	0.35	0.38	
Disagree	0.04	0.06	0.02	0.09	
New HIV/AIDS service facility built in the neighbourhood					
Oppose	0.02	0.08	0.08	0.36	< 0.001^*^
Neutral	0.48	0.63	0.72	0.54	
Support	0.51	0.29	0.20	0.09	
List HIV infection as a statutory notifiable disease					
Oppose	0.46	0.34	0.18	0.13	< 0.001^*^
Neutral	0.27	0.24	0.36	0.21	
Support	0.28	0.42	0.46	0.66	
Provide pre-exposure prophylaxis to anyone who needs it					
Oppose	0.06	0.08	0.06	0.12	< 0.001^*^
Neutral	0.16	0.16	0.25	0.23	
Support	0.78	0.77	0.69	0.65	
Compulsory treatment for people living with HIV					
Oppose	0.35	0.26	0.15	0.11	< 0.001^*^
Neutral	0.29	0.24	0.32	0.22	
Support	0.37	0.50	0.53	0.67	
Provide single-use, sterile syringes to injection-drug users					
Oppose	0.31	0.39	0.29	0.40	< 0.001^*^
Neutral	0.30	0.23	0.36	0.22	
Support	0.39	0.39	0.35	0.38	
Measures to encourage HIV testing among all sexually active people					
Oppose	0.37	0.37	0.30	0.33	0.003^*^
Neutral	0.25	0.22	0.31	0.26	
Support	0.38	0.41	0.39	0.40	
Criminalise sexual activity of people living with HIV					
Oppose	0.88	0.70	0.62	0.48	<0.001^*^
Neutral	0.10	0.21	0.29	0.29	
Support	0.02	0.09	0.09	0.24	

^*^p value< 0.05

Class 1 *“Low stigma”* (37%) was characterised by decreased probabilities of all stigma related indicators. Low level of fear of HIV infection was one of the distinguishing characteristics of this class, including participants who had the lowest probabilities of endorsing misconceptions about HIV transmission (1% were not certain that HIV cannot be transmitted by touching; 44% for kissing, 9% for sharing meals or eating utensils,15% for sharing toilet or swimming pools, and 22% for mosquito bites), expressing fear towards HIV infection (60%) and discomfort in getting along with PLWH (1% in working or attending class; 0% in having social interaction; 3% in having casual physical contact; 65% in developing intimate relationship) as compared to all other classes. This class was also characterised by low level of negative social judgment towards PLWH, as indicated by the lowest probability of agreement with the stereotyped statement that majority of PLWH in Hong Kong are men who have sex with men, illicit drug users, commercial sex workers or their patrons (38%). Low level of concern about socioeconomic ramification of HIV was another distinguishing feature, as reflected by the lowest proportion of members of the class perceiving that PLWH could not live normal lives as other people (0% in working / social life / marriage; 8% in sexual life; 26% in having children). Moreover, experiences of perceived stigma in this group were less common than average (58–79%), despite the overall high percentage reporting such experiences. Regarding the stigma manifestations, this class had the highest share of individuals who agreed that PLWH deserve same level of social support as those with other chronic illnesses (79%). They were the most likely to support policy interventions for protecting PLWH (e.g., 51% supported a new HIV/AIDS service facility built in the neighbourhood) and oppose those reinforcing discrimination (e.g., 46% opposed listing HIV infection as a statutory notifiable disease; 88% opposed criminalising sexual activity of people living with HIV).

Class 2 *“Modest stigma (values-driven)”* (24%) and class 3 *“Moderate stigma (fear-driven)”* (24%) included participants who were more likely to endorse some but not all drivers of HIV-related stigma. HIV-related stigma among *“Modest stigma (values-driven)”* class was driven mainly by high level of concern about socioeconomic ramification of HIV. Participants belonging to this class had higher probabilities to perceive that PLWH could not live normally as other people (9% in working; 16% in social life; 59% in marriage; 96% in sexual life; 95% in having children). HIV-related stigma among members of “*Moderate stigma (fear-driven)” class* was driven mainly by high level of fear of infection, who had elevated probabilities of endorsing misconceptions about HIV transmission (45% could not be certain that HIV cannot be transmitted by touching, 98% for kissing; 81% for sharing meals or eating utensils; 89% for sharing toilet or swimming pools; 75% for mosquito bites). Regarding stigma manifestations, these two classes tended to have more subtle and/or ambivalent stigma experience and practices (e.g., 63% of class 2 and 72% of class 3 indicated neutral response to new HIV/AIDS service facility built in the neighbourhood).

Class 4 *“High stigma”* (15%) stood out with elevated probabilities of all stigma related indicators. Participants from this class had the highest probabilities of endorsing misconceptions about HIV transmission, which was comparable to that of class 3* “Moderate stigma (fear-driven stigma)”*. This class also had the highest probabilities of expressing fear towards HIV infection (95%) and feeling discomfort in getting along with PLWH (74% in working or attending class; 85% in having social interaction; 97% in having casual physical contact; 99% in developing intimate relationship). Stereotyping of PLWH was common in this class (77%). Similar to class 2 *“Modest stigma (values-driven stigma)”*, higher proportion of this class perceived that PLWH could not live normal lives as other people. Probability of agreeing that PLWH deserve same level of social support as those with other chronic illness was the lowest (54%). They were least likely to support policy intervention for protecting PLWH (e.g., 9% supporting new HIV/AIDS service facility built in the neighbourhood), but more likely to support those reinforcing discrimination (e.g., 66% supported listing HIV infection as a statutory notifiable disease; 67% supported compulsory treatment for PLWH; 24% supported criminalising sexual activity of PLWH).

## Factors associated with stigma class membership

Compared with the *“Low stigma”* (class 1—reference group), not knowing someone living with HIV was significantly associated with increased odds of membership in any of the classes endorsing higher level of stigma, with the most prominent effect observed in *“High stigma”***(**Table 5**)**. More specifically, those not knowing someone living with HIV had 1.93 (95% CI 1.34–2.79), 3.71 (95% CI 2.33–5.91) and 5.50 (95% CI 2.77–10.94) fold increased odds of belonging to *“Modest stigma (values-driven)”*, *“Moderate stigma (fear-driven)”* and *“High stigma”* class respectively. Older age (OR = 2.42, 95% CI 1.79–3.26) and being married or cohabiting (OR = 1.56, 95% CI 1.25–1.95) were associated with increased odds of membership in *“High stigma”*. Those aged 35–54 and ≥ 55 were 37% (95% CI 0.52–0.77) and 54% (95% CI 0.35–0.61) less likely to belong to *“Modest stigma (value-driven)”.* Female were 1.39 (95% CI 1.15–1.67) times more likely to belong to *“Moderate stigma (fear-driven)”*. On the other hand, having attained post-secondary education level (*Class 3:* OR = 0.61, 95% CI 0.47–0.80; *Class 4:* OR = 0.52, 95% CI 0.39–0.70) and being economic active (*Class 3:* OR = 0.70, 95% CI 0.57–0.87; *Class 4:* OR = 0.57, 95% CI 0.45–0.73) were associated with decreased odds of membership in *“Moderate stigma (fear-driven)”* and *“High stigma”*.

**Table 5:** Socioeconomic correlates of HIV-related stigma classes membership: Results of multinominal logistic regression models

Reference group—Class 1 *Low stigma*	Class 2 Modest stigma	Class 3 Moderate stigma	Class 4 High stigma
	**Odd ratio (95% CI)**		
**Socioeconomic characteristics**			
**Gender **(reference category: male)			
Female	0.92 (0.76, 1.11)	**1.39 (1.15, 1.67)**	0.94 (0.76, 1.18)
Transgender	3.01 (0.55, 16.48)	0.90 (0.08, 9.98)	#
**Age**(reference category: 18–34)			
35–54	**0.63 (0.52, 0.77)**	**0.79 (0.64, 0.98)**	1.21 (0.92, 1.59)
55 or above	**0.46 (0.35, 0.61)**	1.25 (0.97, 1.61)	**2.42 (1.79, 3.26)**
**Education**(reference category: secondary or below)			
Post secondary	Reference		
	1.19 (0.88, 1.62)	**0.61 (0.47, 0.80)**	**0.52 (0.39, 0.70)**
**Employment status **(reference category: economic inactive (e.g., employed, student, homemaker, retired))			
Employed (any forms)	**1.42 (1.12, 1.81)**	**0.70 (0.57, 0.87)**	**0.57 (0.45, 0.73)**
**Personal monthly income **(reference category: Lower income (i.e., ≤ median personal monthly income))			
Higher	1.20 (0.98, 1.46)	0.84 (0.69, 1.02)	0.81 (0.64, 1.02)
**Ethnicity and residency status **(reference category: Chinese Hong Kong permanent resident)			
Others	0.59 (0.21, 1.67)	0.59 (0.21, 1.67)	0.78 (0.25, 2.41)
**Marital status **(reference category: Single/ divorced/ separated/ widowed)			
Married or cohabiting	0.95 (0.79, 1.14)	1.07 (0.89, 1.29)	**1.56 (1.25, 1.95)**
**Knows someone with HIV**(reference category: yes)			
No	**1.93 (1.34, 2.79)**	**3.71 (2.33, 5.91)**	**5.50 (2.77, 10.94)**

Lack of awareness of U = U (*Class 2:* OR = 1.43, 95% CI 1.17–1.73; *Class 3:* OR = 2.42, 95% CI 1.96–3.00; *Class 4:* OR = 2.73, 95% CI 2.10–3.55) and PrEP (*Class 2:* OR = 1.53, 95% CI 1.22–1.92; *Class 3:* OR = 3.24, 95% CI 2.46–4.26; *Class 4:* OR = 3.53, 95% CI 2.49–4.99) was associated with increased likelihood of belonging to any of the classes with higher level of stigma **(**Table 6**)**. Those who expressed scepticism toward U = U (e.g., “There is still risk of transmission even if the viral load of a person living with HIV is undetectable”; *Class 2:* OR = 1.76, 95% CI 1.42–2.18; *Class 3:* OR = 3.48, 95% CI 2.71–4.46; *Class 4:* OR = 5.70, 95% CI 3.97–8.19) and concerns about anticipated risk compensation related to U = U (e.g. increased condom sex; *Class 2:* OR = 1.50, 95% CI 1.23–1.83; *Class 3:* OR = 2.03, 95% CI 1.64–2.50; *Class 4:* OR = 2.77, 95% CI 2.10–3.64) were more likely to endorse higher level of stigma. Similarly, low level of perceived effectiveness of U = U (*Class 2:* OR = 1.72, 95% CI 1.43–2.08; *Class 3:* OR = 2.00, 95% CI 1.65–2.42; *Class 4:* OR = 2.92, 95% CI 2.29–3.71) and PrEP (*Class 2:* OR = 1.44, 95% CI 1.10–1.88; *Class 3:* OR = 2.12, 95% CI 1.65–2.72; *Class 4:* OR = 2.73, 95% CI 2.07–3.60) in HIV prevention were associated with increased odds of membership in classes with higher stigma level. Generally, those believing that the U = U knowledge made themselves less at ease in getting along with PLWH were more likely to belong to *“High stigma”* and *“Moderate stigma (fear-driven)”*.

**Table 6:** U = U and PrEP perceptual correlates of HIV-related stigma classes membership

Reference group—Class 1 Low stigma	Class 2 Modest stigma	Class 3 Moderate stigma	Class 4 High stigma
	**Odd ratio (95% CI)**		
**Awareness and perception towards U = U and PrEP**			
**Awareness of U = U**(reference category: yes)			
No	**1.43 (1.17, 1.73)**	**2.42 (1.96, 3.00)**	**2.73 (2.10, 3.55)**
**Awareness of PrEP**(reference category: yes)			
No	**1.53 (1.22, 1.92)**	**3.24 (2.46, 4.26)**	**3.53 (2.49, 4.99)**
**Scepticism towards U = U and anticipated risk compensation related to U = U **(reference category: disagree)			
There is still risk of transmission even if the viral load of a person living with HIV is undetectable. **(scepticism with the scientific evidence)**	**1.76 (1.42, 2.18)**	**3.48 (2.71, 4.46)**	**5.70 (3.97, 8.19)**
Even if the viral load of a person living with HIV has reached a very low level, there is no guarantee of future fluctuations that would affect the risk of transmission. **(scepticism with the scientific evidence)**	**1.89 (1.46, 2.46)**	**2.88 (2.14, 3.87)**	**3.41 (2.31, 5.01)**
Whether people living with HIV can maintain U = U status depends largely on medication adherence. **(scepticism with the personal responsibility)**	0.72 (0.51, 1.02)	**0.69 (0.49, 0.97)**	0.74 (0.49, 1.10)
U = U concept could lead to increased condomless sex. **(anticipated risk compensation related to U = U)**	**1.50 (1.23, 1.83)**	**2.03 (1.64, 2.50)**	**2.77 (2.10, 3.64)**
U = U concept could lead to increased number of sex. **(anticipated risk compensation related to U = U)**	**1.54 (1.28, 1.85)**	**2.07 (1.72, 2.51)**	**3.52 (2.78, 4.45)**
**Perceived effectiveness of different strategies in preventing HIV infection **(reference category—61%—100% (a lot of to complete protection))			
Taking pre-exposure prophylaxis	**1.44 (1.10, 1.88)**	**2.12 (1.65, 2.72)**	**2.73 (2.07, 3.60)**
HIV-positive partner taking drugs to maintain undetectable viral load	**1.72 (1.43, 2.08)**	**2.00 (1.65, 2.42)**	**2.92 (2.29, 3.71)**
Using condom for sex	**1.47 (1.22, 1.78)**	**2.04 (1.68, 2.48)**	**2.85 (2.23, 3.64)**
Reduction in number of sexual partners	0.84 (0.69, 1.01)	**0.77 (0.63, 0.93)**	0.89 (0.71, 1.12)
**Perceived impact of U = U in improving comfort level with PLWH **(reference category – no difference)			
Work or attend class with him/her			
More at ease	1.19 (0.97, 1.46)	1.09 (0.88, 1.34)	**1.28 (1.00, 1.64)**
Less at ease	1.23 (0.43, 3.57)	**4.46 (1.97, 10.09)**	**17.17 (7.99, 36.87)**
Make friend with him/her, and have social interactions without physical contact			
More at ease	**1.28 (1.05, 1.56)**	1.17 (0.96, 1.44)	1.11 (0.86, 1.43)
Less at ease	1.12 (0.40, 3.17)	**5.01 (2.34, 10.74)**	**19.86 (9.72, 40.59)**
Have casual physical contact with him/her			
More at ease	**1.24 (1.02, 1.51)**	1.14 (0.94, 1.40)	0.79 (0.61, 1.03)
Less at ease	2.00 (0.89, 4.49)	**7.29 (3.75, 14.20)**	**19.08 (9.98, 36.48)**
Develop intimate relationship with him/her			
More at ease	**0.72 (0.59, 0.88)**	**0.39 (0.31, 0.48)**	**0.11 (0.07, 0.17)**
Less at ease	1.30 (0.96, 1.77)	**1.85 (1.40, 2.45)**	**2.51 (1.87, 3.37)**

## Discussion

Results of this study suggested that awareness of U = U and PrEP was low among the general population in Hong Kong. Perceived effectiveness of U = U and PrEP in HIV prevention, as compared with the conventional strategies such as condom use, was relatively low. Scepticisms towards U = U and concerns about the anticipated risk compensation related to U = U were common. LCA revealed four distinct population classes with different HIV-related stigma profiles (i.e., stigma drivers, experience and practices). While *“Low stigma”* accounted for more than one-third (37%) of respondents, the remainder belonged to *“Modest stigma”* (24%), *“Moderate stigma”* (24%) and *“High stigma”* (15%). The identified stigma classes had unique sociodemographic correlates and significant associations with the awareness and perceptions towards U = U and PrEP. Consistent with previous local studies [10, 11], our findings showed that prevalence of HIV-related stigma was high among the general population in Hong Kong (i.e., about two-third of respondents belonged to subgroups endorsing moderate or higher level of stigma). Delineation of population classes characterised with unique HIV-related stigma profile could potentially inform future stigma reduction efforts, which could be enhanced with tailored interventions for targeted community groups.

The *“High stigma”* class comprised mostly older adults. Individuals from this class were less likely to have attained higher educational level and hold any form of employment. Educational level has been reported to be one of the important factors associated with HIV-related stigma in previous studies [10, 11, 19, 20]. Those who had lower educational attainment were more likely to lack knowledge about HIV and endorse misconception about HIV transmission, resulting in more fear of contagion, stereotypical views as well as discriminatory attitudes towards PLWH [10, 11]. Lack of working experience might limit one’s perspectives and acceptance of social diversity [21], contributing to the high level of HIV-related stigma among this group. The traditional social-cultural values and norms deeply rooted in Chinese older adults could be another reason underpinning the high level of stigma among this group, in which PLWH were associated with violation of social norms, failed filial piety and familial responsibility, as well as loss of face [12–14]. Given that individuals from this class endorsed strongly negative attitudes towards PLWH (i.e. overall elevated probabilities of endorsing all stigma related indicators), they were less likely to benefit from the interventions attempting to make them to think differently and more favourably towards PLWH [22]. Structural interventions like strengthening the legal and policy environment to protect PLWH especially in the work or health care settings, might be required [23]. Re-examination and revision of existing practices which could exacerbate stigmatisation [10] could be important to minimise the public stigma associated with members of this class. HIV prevention interventions targeting male clients of female commercial sex workers often reinforce public’s perception of HIV infection as the result of promiscuity, creating further stigmatisation [10]. Besides, media coverage for HIV surveillance and risk communication in Hong Kong tended to highlight the role of MSM, bisexual man, commercial sex workers and their client as the population groups responsible for the HIV epidemic, perpetuating the negative stereotyping and marginalisation [10].

On the other hand, the *“Modest stigma (value-driven)”* and *“Moderate stigma (fear-driven)”* classes appeared to be more easily amenable to stigma-reduction interventions aiming to cultivate attitudinal and behavioural change, given that they tended to endorse more implicit and ambivalent forms of stigma toward PLWH [24]. The stigma drivers and mechanisms underlying the stigmatisation process were indeed very different between these two classes, despite the similar stigma manifestations. The *“Modest stigma (value-driven)”* class comprised mostly economically active younger people. Compared with other sigma classes, high level of concern about socioeconomic ramification of HIV played a very important role in shaping the stigma in this class. Individuals from this class tended to perceive that PLWH could not live normally as other people and have poor prospects in employment, friendship, romantic relationship and family life. The negative feelings (e.g., hopelessness, disgust, frustration) associated with such perceptions might lead to unwillingness to interact and associate with PLWH as well as avoidance attitude. Media portrayal (e.g., News, TV programme and movies) of HIV, in which PLWH were presented with negative social image compounded by inaccurate and biased information, might be one of the explanations [25]. Intentional media depiction incorporating more comprehensive, accurate, and up-to-date information on HIV prevention and treatment (i.e., TasP, U = U and PrEP) could help to produce more positive and realistic portrayal of HIV. Recall that those not knowing someone with HIV were more likely to belong to population classes endorsing higher level of HIV-related stigma, social contact interventions could be useful. Message framing by sharing stories of PLWH and adding a “human face” to the message could convey more realistic and humanising image, which might help to reduce the stigma among this group [20, 23]. While this class comprised mainly economic active younger people, making good use of social media (e.g., Facebook, Instagram) which allow reposting and re-dissemination of contents could enhance the penetration of HIV campaigns [26]. The *“Moderate stigma (fear-driven)”* class comprised mostly females. Similar to *“High stigma”*, individuals from this class were less likely to have higher educational level and hold any form of employment. Fear of infection due to misconception of HIV transmission appeared to play a more important role in shaping the HIV-related stigma. While HIV disproportionately affects men who have sex with men (MSM) and bisexual men in most developed settings such as Hong Kong, women in the general population are less likely to be targeted in the HIV educational programme or receive HIV-related information from the health care professionals. In this connection, they might develop their own misconceptions on HIV transmission. Educational interventions focusing on HIV transmission might be useful to replace the myths and thus reduce the stigma in this class [23].

Overall, our study showed that people who lacked awareness of and/or had low confidence in the role of U = U and/or PrEP in HIV prevention were more likely to belong to population classes endorsing higher level of HIV-related stigma. Our findings echo previous research suggesting the potentials of U = U in dismantling HIV-related stigma among the general population [27, 28]. In Hong Kong awareness of U = U was generally low, enhanced efforts for universal dissemination of U = U is necessary. However, uncritical promotion and celebration of U = U knowledge without carefully considering how the message is received, interpreted and internalised might create unintended divisions between HIV positive and negative individuals and exacerbate existing stigma [29–31]. In some population groups, the high level of scepticism towards U = U, considerable concerns about the anticipated risk compensation related to U = U, and low perceived effectiveness of U = U in HIV prevention highlighted the need for interventions to improve its acceptance. Tailored U = U messages, which consider differing capacities for health literacy (e.g., one’s ability to elaborate on a message in a given context) and one’s HIV-related stigma profile, could be an important strategy [28]. Further research examining effectiveness of differentially framing U = U messages tailored to populations with different stigma profiles is warranted. Furthermore, education and training, as well as clear and concise guidelines are needed to support healthcare professionals for accurate and impactful communication [29]. Meanwhile, creating supportive environment which ensures universal access to HIV testing, treatment and care is necessary to facilitate the general public in their acceptance and adoption of U = U knowledge. Given that current PrEP promotion / education generally focuses on key populations at substantial risk of HIV infection such as MSM, it is not surprising that the awareness and confidence towards PrEP has been slow in the general population in Hong Kong. Our finding suggested that expanding PrEP promotion / education in general population might be useful to reduce the HIV-related stigma. However, such expansion has to carefully balance the risk of increasing PrEP-associated stigma which is a major barrier to PrEP update and adherence [32].

This study has several limitations. First, the use of self-reported data may be subjected to social desirability bias. Some respondents might provide socially desirable responses, which could possibly lead to an underestimation of the level of HIV-related stigma. Second, since data was not available from those invited to participate but chosen not to respond, study results might suffer from non-response bias. Third, while stigma is a complex and multidimensional phenomenon which involves many constructs with interactions between constructs, these might not have been sufficiently captured in this study. Fourth, the cross-sectional nature of the survey could not allow for causal inference and assessment of the temporal change in stigma drivers and manifestations. Firth, the survey sample might not be perfectly representative of the general population of Hong Kong since it tended to be younger and better educated. Finally, extrapolation of results to settings with different cultural backgrounds should be cautioned. Notwithstanding these limitations, the present study was a unique attempt to quantitatively characterise the patterns of HIV-related stigma among a large representative sample of general population in a metropolitan city in Asia Pacific. Furthermore, our study had provided an updated profile of the community’s knowledge, awareness and perception about HIV transmission, prevention and treatment in the era of U = U and PrEP.

## Conclusion

In general, the prevalence of public stigma towards PLWH in Hong Kong is high. Lack of awareness and confidence towards the biomedical advances in HIV such as U = U knowledge and PrEP was common. Analysis of community patterns of HIV-related stigma revealed the diversity among population subgroups regarding their stigma drivers and manifestations, and explore the role of socio-demographics, awareness and perception of U = U and PrEP in impacting their stigma endorsement. Incorporating such knowledge in the development and implementation of more appropriately tailored interventions could be important to enhance future stigma reduction efforts.

## Data Availability

Data is provided within the manuscript an the supplementary information file.

## References

[1] Cohen MS, Chen YQ, McCauley M, Gamble T, Hosseinipour MC, Kumarasamy N (2016). Antiretroviral therapy for the prevention of HIV-1 transmission. N Engl J Med.

[2] Rodger AJ, Cambiano V, Bruun T, Vernazza P, Collins S, Degen O (2019). Risk of HIV transmission through condomless sex in serodifferent gay couples with the HIV-positive partner taking suppressive antiretroviral therapy (PARTNER): final results of a multicentre, prospective, observational study. Lancet.

[3] Bavinton BR, Pinto AN, Phanuphak N, Grinsztejn B, Prestage GP, Zablotska-Manos IB (2018). Viral suppression and HIV transmission in serodiscordant male couples: an international, prospective, observational, cohort study. Lancet HIV.

[4] McCormack S, Dunn DT, Desai M, Dolling DI, Gafos M, Gilson R (2016). Pre-exposure prophylaxis to prevent the acquisition of HIV-1 infection (PROUD): effectiveness results from the pilot phase of a pragmatic open-label randomised trial. Lancet.

[5] Goffman E (1963). Stigma: notes on the management of spoiled identity.

[6] Link BG, Phelan JC (2001). Conceptualizing stigma. Annu Rev Sociol.

[7] Herek GM, Capitanio JP (1999). AIDS stigma and sexual prejudice. Am Behav Sci.

[8] Herek GM (1999). AIDS and stigma. Am Behav Sci.

[9] Lo A, Wan S. Hong Kong STD/AIDS update: a quarterly surveillance report. Vol.29 No.1, Quarter 1 – 2023. Hong Kong: Centre for Health Protection, Department of Health; 2023. Available from: https://www.chp.gov.hk/files/pdf/std23q1.pdf. Cited 2023 Aug 5

[10] Yeo TED, Chu TH (2017). Social-cultural factors of HIV-related stigma among the Chinese general population in Hong Kong. AIDS Care.

[11] Lau JT, Tsui HY (2005). Discriminatory attitudes towards people living with HIV/AIDS and associated factors: a population based study in the Chinese general population. Sex Transm Infect.

[12] Yang LH, Kleinman A (2008). 'Face' and the embodiment of stigma in China: the cases of schizophrenia and AIDS. Soc Sci Med.

[13] Zhou YR (2006). Homosexuality, seropositivity, and family obligations: perspectives of HIV-infected men who have sex with men in China. Cult Health Sex.

[14] Lau JT, Choi KC, Tsui HY, Su X (2007). Associations between stigmatization toward HIV-related vulnerable groups and similar attitudes toward people living with HIV/AIDS: branches of the same tree?. AIDS Care.

[15] Stangl AL, Earnshaw VA, Logie CH, van Brakel W, Simbayi LC, Barré I, Dovidio JF (2019). The health stigma and discrimination framework: a global, crosscutting framework to inform research, intervention development, and policy on health-related stigmas. BMC Med..

[16] Stangl AL, Brady L, Fritz K (2012). STRIVE technical brief: measuring HIV stigma and discrimination.

[17] Linzer DA, Lewis JB (2011). poLCA: an R package for polytomous variable latent class analysis. J Stat Softw.

[18] Lanza ST, Rhoades BL (2013). Latent class analysis: an alternative perspective on subgroup analysis in prevention and treatment. Prev Sci.

[19] Li X, Yuan L, Li X, Shi J, Jiang L, Zhang C (2017). Factors associated with stigma attitude towards people living with HIV among general individuals in Heilongjiang, Northeast China. BMC Infect Dis.

[20] Pharris A, Hoa NP, Tishelman C, Marrone G, Kim Chuc NT, Brugha R (2011). Community patterns of stigma towards persons living with HIV: a population-based latent class analysis from rural Vietnam. BMC Public Health.

[21] Staff J, Messersmith EE, Schulenberg JE, Lerner RM, Steinberg L (2009). Adolescents and the world of work. Handbook of adolescent psychology: contextual influences on adolescent development.

[22] Howe LC, Krosnick JA (2017). Attitude strength. Annu Rev Psychol.

[23] Cook JE, Purdie-Vaughns V, Meyer IH, Busch JTA (2014). Intervening within and across levels: a multilevel approach to stigma and public health. Soc Sci Med.

[24] Jonas K, Ziegler R, Hewstone M, Schut H, de Wit JBF, van den Bos K, Stroebe MS (2007). Attitudinal ambivalence. The scope of social psychology: theory and applications.

[25] Mo PKH, Ng CTY (2017). Stigmatization among people living with HIV in Hong Kong: a qualitative study. Health Expect.

[26] Taggart T, Grewe ME, Conserve DF, Gliwa C, Roman IM (2015). Social media and HIV: a systematic review of uses of social media in HIV communication. J Med Internet Res.

[27] Rivera AV, Carrillo SA, Braunstein SL (2021). Prevalence of U = U awareness and its association with anticipated HIV stigma among low-income heterosexually active black and Latino adults in New York City, 2019. AIDS Patient Care STDs.

[28] Coyne R, Walsh JC, Noone C (2022). Awareness, understanding and HIV stigma in response to undetectable = untransmittable messages: findings from a nationally representative sample in the United Kingdom. AIDS Behav.

[29] Grace D, Stewart M, Blaque E, Ryu H, Anand P, Gaspar M, Worthington C, Gilbert M (2022). Challenges to communicating the Undetectable equals Untransmittable (U=U) HIV prevention message: healthcare provider perspectives. PLoS One.

[30] Tan RKJ, Lim JM, Chan JKW (2021). Is “Undetectable = Untransmissible” good public health messaging?. AMA J Ethics.

[31] Grace D, Nath R, Parry R, Connell J, Wong J, Grennan T (2021). '… if U equals U what does the second U mean?': sexual minority men's accounts of HIV undetectability and untransmittable scepticism. Cult Health Sex.

[32] Golub SA (2018). PrEP stigma: implicit and explicit drivers of disparity. Curr HIV/AIDS Rep.

